# Lived experiences of women who survived from pre-eclampsia and eclampsia in public hospitals of Shashemene Town, Oromia Region, Ethiopia: a qualitative study.

**DOI:** 10.12688/f1000research.129648.3

**Published:** 2025-04-07

**Authors:** Negeso Gebeyehu, Aman Urgessa, Daniel Yohannes, Aster Yalew, Muluneh Ahmed, Meron Admasu

**Affiliations:** 1 Midwifery, Madda Walabu University, Shashemene Campus, Shashemene, Oromia, 264, Ethiopia; 2 Nursing, Madda Walabu University, Shashemene Campus, Shashemene, Oromia, 264, Ethiopia; 3 Nursing, Wachemo University, Durame Campus, Durame, SNNPR, 640, Ethiopia

**Keywords:** Experiences, preeclampsia, eclampsia, survivors, Shashemene

## Abstract

**Objective:**

The main purpose of this study was to explore experiences of women who survived pre-eclampsia and eclampsia in Shashemene referral hospital and Melka Oda general hospital, 2021.

**Design:**

An institution-based exploratory qualitative study with a descriptive phenomenological study design

**Setting:**

The present study was conducted in Shashemene referral hospital and Melka Oda general hospital from March 03 to May 18, 2021.

**Participants:**

A total of 17 individual in-depth interviews (IDIs) were conducted with women who experienced and survived pre-eclampsia and eclampsia. Data were analyzed through thematic analysis using Atlas-ti software.

**Result:**

The present study revealed that women’s level of awareness on raised blood pressure and or convulsion occurring during pregnancy was poor. Barriers that limit pregnant women from getting treatment at the earliest point included misconception, lack of insight, failure to accept counseling from health care providers, low income, and influence from husbands. Discussions with the women in this study showed that these mothers were not counseled on danger signs during antenatal care check-ups. Almost all of the women were very concerned and frustrated due to their diagnosis.

**Conclusions:**

Women’s awareness of pre-eclampsia and eclampsia was poor. Improving awareness of hypertensive disorders in pregnancy and enhancing the quality of antenatal care is essential.

## Strengths and limitations of the present study

▪ As far as our knowledge is concerned, this the first study in Ethiopia investigating the lived experiences of women who have survived pre-eclampsia and eclampsia.
▪ The study design employed which is a qualitative study design enabled a thorough investigation of lived experiences of women who survived pre-eclampsia and eclampsia in public hospitals of Shashemene Town.
▪ The interviews were done in Afan Oromo and Amharic, the participants’ native language; a thorough translation process was used to make sure that no meaning was lost.
▪ Selection bias exists due to the fact that women were recruited only from hospitals.

## Introduction

Pre-eclampsia is a multi-system disorder, specific to pregnancy, that is associated with raised blood pressure (≥140 mmHg/90 mmHg) and proteinuria which usually occurs after 20 weeks of gestation. Eclampsia is one or more convulsions in association with the syndrome of pre-eclampsia. ^1^ Worldwide, pre-eclampsia complicates 2% to 8% of pregnancies whereas, in resource-poor countries, estimates of the incidence of eclampsia vary from 1/100 to 1/1700 deliveries. ^1^

Pre-eclampsia and eclampsia are associated with increased complications including placenta abruption, preterm birth, fetal growth restriction acute renal failure cerebrovascular and cardiovascular complications, disseminated intravascular coagulation, and maternal death. ^2^

Evidence shows that in Sub-Saharan African (SSA) countries, pre-eclampsia and eclampsia are among the top five leading causes of morbidity and mortality of women and neonates. Consequently, SSA countries experience the highest maternal and newborn mortality. ^3^^,^^4^

Despite efforts made to reduce maternal mortality in Ethiopia, there is still a high burden of maternal death with pre-eclampsia and eclampsia being major contributing factors. ^5^^,^^6^ It is crucial to comprehend the sociocultural and behavioral aspects of pregnant women who have survived preeclampsia and eclampsia. Hence, the findings from this study could be used in designing appropriate interventions in handling bottlenecks related with these women.

Early diagnosis and management can help to reduce the dangers of pre-eclampsia and eclampsia and their complications; the majority of deaths related to this condition are avoidable when care is given at the right time. Avoiding delays and hindrances that are currently occurring in diagnosis and management are critical in this regard. ^7^

To the best of our knowledge, so far, studies have not been conducted to explore experiences of women survived from pre-eclampsia and eclampsia in Ethiopia. Previous studies merely focused on prevalence and factors associated with pre-eclampsia. ^8^^,^^9^

Therefore, this study aimed to explore lived experiences of women who survived pre-eclampsia and eclampsia in Shashemene referral hospital and Melka Oda general hospital – the two public hospitals in the town. It identifies the barriers and enabling factors that affect their healthcare-seeking behaviors, and it describes ideas raised by these women to overcome these challenges.

## Methods

### Study setting and design

An institution-based exploratory qualitative study with a descriptive phenomenological study design was carried out from March 03 to May 18, 2021, at the two public hospitals of Shashemene town: Shashemene referral hospital and Melka Oda general hospital. Shashemene referral hospital is found in Shashemene town which is situated 238 km from Addis Ababa and nine Kms North of Shashemene town and Melka Oda general hospital, which is located in Shashemene town, 250 km away from the capital city, Addis Ababa.

*Sample size determination*

The sample size was estimated using previous methodological research ^10^ conducted on similar topic, the level of information saturation, and the diversity of ideas among the participants. As a result, a total of seventeen individual in-depth interviews (IDIs) were conducted with women who experienced and survived pre-eclampsia and eclampsia.

### Participants

The source populations were all women who suffered and survived from pre-eclampsia and eclampsia and were discharged from the hospitals. The study population encompassed all purposively selected women who suffered and survived from pre-eclampsia and eclampsia and were discharged from the hospitals.

Women who suffered and survived from pre-eclampsia and eclampsia and were discharged from the hospital were included in the present study whereas women who were not willing to participate in the study were excluded.

### Data collection

Purposive sampling technique was employed to approach study participants. Individual in-depth interviews (IDIs) were conducted with women who suffered from pre-eclampsia and eclampsia, survived, and were discharged from the hospital. Information on socio-demographic characteristics, pregnancy, delivery, postnatal experiences, antenatal care (ANC), knowledge on pre-eclampsia and eclampsia, and factors that affect care-seeking at individual, household, community, and health systems levels were collected using a semi-structured interview guide. Instruments were pre-tested in Negelle Arsi Primary Hospital by authors to ensure that appropriate questions are being asked and that questions do not make respondents uncomfortable and/or confused because of combining two or more important issues in a single question. The pretest was approved by Institutional Review Board (IRB) of the University. Two people attended each interview; one conducted the interview and the other took field notes. Criterion followed while note taking include but not limited to taking basic information like study tile, PI of the study, season and date of data collection, geographic setting, demographics of the study participants, and setting. To maintain the integrity of the participants; from the very beginning, sufficient information was provided to study participants on the aim of the study, and they were also told that the information they offer will be kept confidential.

Semi-structured interview guides used in the present study were taken from a similar study conducted in Nigeria which were validated. ^10^ Interview guide questions were prepared first in English, then translated to Afan Oromo, and Amharic and translated back to English to check for consistency by expert (health professional) who is familiar with both local languages and English. Thus, a thorough translation process was used to make sure that no meaning was lost. There were no changes made after the translation is made. 40–50 minutes were spent on the interview. Confidentiality was stressed in order to foster a relaxed atmosphere where participants may divulge more personal information and give an in-depth account of their experiences. Open ended questions and probing were used to extract additional information. During the data collection period, supplementary notes were taken through the non-participatory observation technique for non-verbal response and the context of the interview. The tape-recorded audio was checked for completeness and field notes were expanded soon after the end of each interview.

To assure the credibility of the study, the data collection process was completed at the data saturation point when no new information was obtained. Both the audio recorder and the field notes were used to collect data, and this enabled cross-checking. NG, AU and DY were data collectors. Due to the fact that non-verbal replies are just as significant as verbal responses, this technique allowed us to ensure credibility of the study.

Moreover, to assure dependability, the present study has clearly described the process selection of study participants, the process of data collection procedure, and data analysis in the method section. To maintain transferability, the finding of the data was precisely described with sufficient details, and a thick description of data was provided by the principal investigator and co-investigators. Lastly, to assure conformability, the audio and field note transcripts were compared and contrasted with the final research report so that to ensure consistency with original data.

### Analysis

Data were analyzed by Atlas-ti software by applying thematic analysis. Researchers who want to reproduce our analysis and have no access for Atlas.ti (since Atlas.ti proprietary software) can use Qualcoder which is the best alternative. After listening repetitively to the audio record of individual IDIs, it was then transcribed verbatim into Afan Oromo and Amharic. The Afan Oromo and Amharic transcripts were then translated to the English language for analysis and made it in line with the interviewers’ field notes. The Afan Oromo, Amharic and English data were cross-checked for accuracy and completeness of the translation before analysis. To ensure that the participants’ identities could not be deduced from the transcripts, they were anonymized. All the transcripts were cautiously and repetitively read to become familiar with it. Codes were developed to extract meanings out of texts and were defined based on contained information, and central meaning. DY verified consistency after NG and AU completed the initial code. The researchers have established a consensus regarding the codes and have clarified intercoder discrepancies. Then, codes were taken to build emerging themes. Inductively, the codebook was created while going over the transcripts. The themes were generated in accordance with the study’s aim by summarizing the codes and organizing them into logical groups with descriptive data segments. The findings were displayed as themes along with exact quotes that provided support. The researchers were not instrumental and did not contribute their own viewpoints in any degree of interpretation. The translated primary documents, quotes, codes and interview guide questions are all available in our underlying and extended data.

## Result

### Socio-demographic & obstetrics characteristics of participants

A total of 17women were participated in this study. The age of participants ranged from 18-35 years (median age: 25 years). The number of children that the women had ranged from one to six. The median age of women at first birth was 19 years. Regarding educational status, three didn’t attend formal education, eight completed primary education, four secondary primary education, one has a diploma, and one has a BSc degree. A total of nine study participants resided in urban while eight resided in rural areas. Sixteen women had attended ANC check-ups during their current pregnancy. A total of four individuals had faced complications in their previous pregnancies. Of the women who had faced complication in their previous pregnancies, three women had pre-eclampsia and eclampsia previously while one woman had experienced antepartum hemorrhage. A total of five women had eclampsia while 12 had pre-eclampsia in the current pregnancy ( Table 1).

**Table 1.** Socio-demographic & obstetrics characteristics of participants.

Variable	Category	Frequency (n=17)	Percent
Age	18-24	7	41.2
	25-34	8	47.1
	≥35	1	5.9
	I don’t know	1	5.9
Educational status	No formal education	3	17.6
	Primary education	8	47.1
	Secondary education	4	23.5
	Diploma	1	5.9
	BSc & above	1	5.9
Place of residency	Urban	9	52.9
	Rural	8	47.1
ANC check-up	Yes	16	94.1
	No	1	5.9
Previous obstetrics complications	Pre-eclampsia & Eclampsia	3	17.6
	Antepartum hemorrhage	1	5.9
	No history of obstetrics complication	13	76.5
Type of hypertensive disorders in pregnancy currently developed	Pre-eclampsia	12	70.6
	Eclampsia	5	29.4

### Emerged themes

The emerged themes in the current study include antenatal care services received and its quality, awareness of raised blood pressure and or convulsion during pregnancy, symptoms experienced, and quality of care received, women’s reaction towards the diagnosis, barriers, and suggestions made by the women to improve health care services received by those who developed pre-eclampsia and eclampsia.

### Antenatal care service offered and its quality

Antenatal care is a maternal healthcare service provided by skilled health professionals to pregnant women. It is provided throughout pregnancy to ensure the best health outcomes for both the mother and neonates. The narratives of the women interviewed showed that blood pressure measurement, urine test, blood test, tetanus toxoid vaccination, and ultrasound were services received at the antenatal care unit. Others, though, disclosed that they were not provided with the aforementioned services.

One of the study participants explained the service she received as follows:

*“…they have measured my BP and weight. I have taken* tetanus toxoid *vaccination” (Participant 16, 20-year-old woman).*

Another participant expressed that tablets for anemia and tetanus toxoid vaccination were given at the ANC unit.

*“They gave me tablets to prevent the occurrence of anemia. They also gave me vaccination here on my arm” (Participant One, 18-year-old woman).*

Another participant said that only a blood test was done. She expressed that the health professionals simply examined her belly.

*“They did nothing. They simply examined me with their hands and sent me here (hospital). However, they have tested my blood” (Participant Two, 22-year-old woman).*

One of the women stated that nothing had been offered to her while she attended ANC check-ups.

*“They didn’t do a urine test and also not measured my blood pressure” (Participant 5, 35-year-old woman).*

Counseling about danger signs is so vital issue that will enable pregnant women to come to a health facility at the earliest point when they develop the symptoms. Hence, complications can be reduced significantly. ^10^ In this regard, most of the women said that they were not counseled for danger signs while they attend ANC check-ups during their current pregnancy.

*“They didn’t tell me these signs [danger signs]. I went for check-ups because I have some headaches. What enabled me to go to the health facility early was the experience I had during the last pregnancy. Unfortunately, they told me that it was common cold & come back home. I went to the health facility because I have fear that some complications might happen. This fear was due to complications that occurred during the last pregnancy. Nobody told me these danger signs” (Participant One, 18-year-old woman).*

Another participant also shares the above experience in which she said that she wasn’t told the danger signs in detail.

*“They did not inform me that [danger sign]. They didn’t tell me in in detail. They simply told me to come back” (Participant 3, 18-year-old woman).*

One of the study participants who developed eclampsia said that the health professional had not counseled her on danger signs.

*“They informed me nothing. I even asked the doctor if I come back for the check-up. The doctor told me that there is no need to come back. He (doctor) told me even I can give birth with no complication” (Participant Four, 30-year-old woman).*

The other participant expressed that one should go repeatedly to receive counseling on danger sign

*“The health professionals have informed me nothing. But, to have such information one should go repeatedly. I went only once and therefore I didn’t get the chance of receiving such counseling” (Participant Five, 35-year-old woman).*

Similarly, the other woman stated that she got the information from her previous experience. However, she said that she is not counseled during her current pregnancy.

*“They didn’t inform me this at all. I had this information during my first pregnancy. Because I suspected hypertension, I was the one who insist to check my BP. When I told them, I have suspected hypertension in the current pregnancy because I had it before, they overlooked my concern and said you will not develop hypertension for you had it in the past” (Participant Seven, 25-year-old woman).*

Another woman stated that she has received counseling on danger signs. She listed the danger signs she was counseled on.

*“Yes, they have informed me…… Bleeding through the vagina before childbirth and also the passage of white discharge before the birth of the baby. They told me to come to the health facility as soon as possible if I see these signs”*

*(Participant Six, 17 year-old woman).*

One participant also stated that she was counseled for danger signs, and she has also listed the signs.

*“Yes, they have informed me. For instance, if the baby is not moving well, its weight is increasing rapidly, swelling of the body, they told me to come to a health facility as early as possible” (Participant 13, 29 year-old woman).*

Women were requested to give opinions on the quality of antenatal care services they received. One woman stated that the health professional didn’t evaluate her thoroughly and they “disrespected” her.

*“All professionals here (at the hospital) disrespected me. They didn’t evaluate me thoroughly. They didn’t serve patients properly” (Participant 13, 29 year-old woman).*

Participant 16 also raised that the health professionals have no extensive experience and said they don’t treat mothers with respect. She also mentioned a lack of ultrasound equipment as a significant problem.

*“The shortcomings are: the facility is not neat, the doctors have no experience, and they didn’t treat mothers with respect, they don’t have an ultrasound. Ultrasound is available everywhere even at the level of kebeles [Kebele is the lowest administrative unit as per the context of study setting]. So, the lack of ultrasound is a significant problem. Health professionals working at the delivery unit are better in terms of the experience they have and the respectful care they provide. Health professionals working at the ANC unit disrespect women. They have no extensive experience. I am not happy with their work” (Participant 16, 20-year-old woman).*

One of the study participants stated that the health professional working at the antenatal care clinic didn’t inform her as her blood pressure increased.

*“There (at ANC), they have checked my blood pressure. But they didn’t inform me that my BP is increasing and the stage of the disease. Otherwise, they have checked me thoroughly” (Participant 13, 29-year-old woman).*

One other participant shares a similar idea to the above, in which she stated that the health professional didn’t inform her as her blood pressure was rising.

*“They did give me drugs. They did not inform me that my blood pressure is rising. When I come back in the third month, they told me that I had raised blood pressure. I was shocked at that moment. Finally, they transferred me to labour ward” (Participant 11, 25-year-old woman).*

One of the women said that the lack of ultrasound was the shortcoming she noticed.

*“The shortcoming is lack of ultrasound. They didn’t check me by ultrasound [at the health center]. Here [at hospital], they checked by ultrasound and said the baby have malformation on its head. Here [at hospital], they said why you just stayed all these seven months. If they had an ultrasound, I wouldn’t have stayed all these months. So, the shortcoming is lack of ultrasound” (Participant Seven, 25-year-old woman).*

However, one of the women stated that she received the right ANC check-up.

*“They had given me the right service. They told me that the gestational age is seven months and two weeks in line with the information here (hospital)” (Participant Five, 35-year-old woman).*

### Symptoms experienced and quality of care received

Study participants developed a wide range of symptoms including headaches, heartburn, loss of consciousness, epigastric pain, blurring of vision, fever, swelling face, and feet.

One of the study participants said she had a headache and blurring of vision.

*“I had a headache. I had also “qaanqee” before the headache [qaanqee is local Afan Oromo language term which means a blurring of vision]. I feel as if something has blown into my eyes. I had this symptom occasionally” (Participant One, 27-year-old woman).*

This participant also stated that she had lost consciousness. She remembered the moment as follows:

*“During that time, I was sleeping. During the early morning I lost my consciousness. My families brought me here tell me that I have oral secretions. I came here by ambulance and finally gave birth” (Participant One, 27-year-old woman).*

Another participant stated that she was experiencing epigastric pain and blurring of vision. This woman also lost her consciousness.

*“I had epigastric pain. After a moment, I developed a blurring of vision. Even before I had blurring of vision, I felt like seeing an object in a mirror. After I experienced these symptoms, I came here……. I lost my consciousness right after I arrived here” (Participant Four, 30 year-old woman).*

One of the women stated that she has developed fever, swelling face, and feet.

*“I had a headache from the moment I develop fever and swelling of face and feet. The health professional told me that the swelling of feet has no significant impact and be vigilant on the swelling of face” (Participant Five, 35-year-old woman).*

The other woman said that she developed fever, her feet swollen, and had heartburn.

*“I went there (health center) because I have an appointment. However, I had a fever. When I splash water on my body, it will be resolved. But, a few minutes later, it will start. My feet were swollen, and I had also heartburn. Then, I requested them to check my BP. When they check it, it was very high. Finally, they referred me to this hospital” (Participant Seven, 25-year-old woman).*

However, one of the study participants said that she had no symptoms at all.

*I don’t have these symptoms [blurring of vision, raised BP, headache] at all. They told me to watch these symptoms. I am waiting for these symptoms. But no such symptoms till now. If they told me I have raised blood pressure, my option is to accept their opinion (Participant 13, 29-year-old woman).*

Study participants expressed the treatment they received after they arrived at the hospital. One of the women stated that health professionals were following her closely and administered drugs.

*“They are following me closely and giving me drugs” (Participant 12, 27 year-old woman).*

Other participants also stated satisfaction with the care given.

*“I am satisfied with the care given” (Participant Eight, 30-year-old woman).*

Another study participant expressed that she received satisfactory care.

*“Well, I have received satisfactory care here. They saved my life here with the help of God. The health professionals here saved my life with the help of God, and I am so excited” (Participant Four, 30-year-old woman).*

The other participant stated that her symptoms resolved after she received treatment at a hospital.

*Right after they gave me injections, I am fine. I have taken also pills and my headaches are gone (Participant Six, 24-year-old woman).*

### Women’s reaction after informed of the diagnosis

Different reactions were observed when women were told they have hypertension. Several women were shocked, frustrated, anxious, and depressed when they heard their diagnosis.

One of the women stated that she was shocked when she was told she had raised blood pressure.

*“I was very shocked. I was having headache & blurring of vision at that moment. I was much stressed. Yesterday, they brought me here because I was very sick. But God gave me this child” (Participant 10, 20-year-old woman).*

The other woman was also concerned when they told her she has hypertension.

*“I said to myself, are they again to terminate this pregnancy as before? I was shocked and disturbed. When they saw me as I am very worried, they counseled me not to be worried and told me that the baby will be born soon. They told me that the pregnancy has to be terminated. By counseling me thoroughly, they calm me” (Participant 11, 25-year-old woman).*

The other study participant also said that she was concerned when they tell her she has hypertension. Besides, she said hypertension is so dangerous.

*“I know that raised blood pressure is dangerous. I was concerned. Then, I calmed down when they gave me drugs and told me that my BP is dropping” (Participant 13, 29-year-old woman).*

One of the women expressed that it is so difficult to have raised blood pressure and it will also result in depression.

*It’s so difficult to have raised blood pressure. The headache and swelling are so difficult. It will result in depression (Participant 17, 22-year-old woman).*

### Women’s awareness of hypertensive disorders occurring during pregnancy

Women’s level of awareness on raised blood pressure and or convulsion occurring during pregnancy was modest and poor. Most of the women (82.35%) were not aware that pregnant women may suffer from raised blood pressure and convulsions.

One of the women said she hadn’t heard of raised blood pressure in pregnant women.

*“I didn’t hear this raised blood pressure on pregnant women. This raised blood pressure occurs in mothers who stopped giving birth. I heard when neighbors say this woman has hypertension. But I didn’t see a pregnant woman who suffered & died from raised blood pressure. This raised blood pressure usually occurs in mothers who stopped giving birth. We need to restrain from consuming meals with high salt, black coffee, and stress. I didn’t see a pregnant woman who suffered from raised blood pressure” (Participant Eight, 30-year-old woman).*

Many of the other study participants didn’t know about raised blood pressure and convulsion occurring during pregnancy.

*“…I don’t know” (Participant 11, 25-year-old woman).*

However, one of the study participants mentioned the symptoms of raised blood pressure.

*“They said women who have raised blood pressure can’t stand, have headache on one side and blurring of vision. But I have not experienced this problem previously” (Participant 15, 26 year-old woman).*

Most of the women stated that raised blood pressure can cause maternal and fetal death. One of the women said that if raised blood pressure is left untreated it will cause hypertension and cardiac disease in the long run.

*“She [the mother] may develop hypertension and cardiac disease later on. The baby weight might be low and can be exposed for different diseases. Even they [both the mother & baby] might die” (Participant 16, 20-year-old woman).*

One of the study participants expressed that the effect of raised blood pressure and convulsion may escalate to the level of death

*“…to the extent of death” (Participant 15, 26-year-old woman).*

One of the women said that staying at home may cause death.

*“They need to check their blood pressure at a health center or a private clinic. If they simply stayed home, they may die. In the rural, they say it’s “Daataa” [A Local Afan Oromo Language which means blood which shaded during childbirth] and encourage women to stay home” (Participant 3, 18-year-old woman).*

Other women didn’t know the fate of a woman who was left untreated for raised blood pressure and/or convulsion.

*“I don’t know what happens to these women” (Participant 8, 30-year-old woman).*

### Barriers to receiving treatments from health facilities

Study participants listed a wide range of barriers that limit pregnant women who developed raised blood pressure and or convulsion from getting treatment at the earliest point. These include misconception, lack of knowledge, fear of keeping children at home alone, low income, unsupportive husband, criticizing & blaming, and failure to be counseled by healthcare providers.

### Misconceptions

Women’s narratives suggested the presence of misconception in society on hypertensive disorders occurring during pregnancy. For instance, swelling of the face & feet during pregnancy is thought to be normal. They believe that blood is accumulated in the face & feet till childbirth, and hence they are swollen. According to the local language, this process is termed *“Daataa”.* The other misconception which was mentioned by the women was dryness of the head a responsible phenomenon for headache. Women also mentioned that these kinds of misconceptions are very risky as they may cost their lives.

One of the women stated this as follows:

*“They [some of the women] believe that swellings of face & feet are a normal event. These swellings occur during the stage of childbirth. Until childbirth, the blood stays in the feet and face, and they name it “Daataa”. People in rural areas believe that severe headache is due to dryness of the head. Because of these, misconception among mothers will cost their lives. It is very important to check blood pressure. Not only mothers, but it will also be better if all individuals check their blood pressure. If you take my situation, firstly, I came here for leakage of fluid. However, as days passed, I developed raised blood pressure. So, if I were at home, I would have missed the opportunity of knowing of the problem” (Participant Eight, 30-year-old woman).*

The other woman also shares the idea of the above mother.

*“There is a misconception in the society. For instance, when I suffered from hypertension during the last pregnancy, my face and legs were swollen. Society perceives that the swollen face and legs are due to blood during pregnancy. They told me it was “daataa” and that’s why you are gaining weight” (Participant One, 27-year-old woman).*

### Lack of knowledge

Women reported a lack of knowledge to be the barrier to receiving adequate care. Sufficient knowledge of pre-eclampsia & eclampsia contributes significantly to its prevention, management, and treatment. Knowledge of the patient’s illness has significant benefits in terms of treatment compliance and helps reduce illness-related complications. One of the women said that lack of knowledge is a barrier to not getting early treatment.

*“It’s lack of knowledge. If she has sufficient knowledge on hypertension, she will go to a facility to get the necessary treatment. Nobody wants to suffer from disease” (Participant Five, 35-year-old woman).*

The other woman also supported the above idea.

*“It’s lack of insight. If you lack insight, you will face complications” (Participant Nine, 20-year-old woman).*

One of the women said that lack of insight and failure to accept counseling of health professionals are factors that limit women.

*“I think it is because they have no insight and failure to accept counseling from health professionals” (Participant 11, 25-year-old woman).*

The other study participant shared a similar idea and additionally stated that there is criticizing and blaming.

*“Lack of insight on this issue. Some mothers might say rather than going to a health facility it is better to gather information from women in the community. This is because they lack knowledge. There is also criticizing and blaming each other” (Participant 16, 20-year-old woman).*

### Poor financial status and an unsupportive spouse

Women mentioned a lack of husband support to be responsible for not getting treatment. Men’s role as a major decision-maker and a major supplier of economic resources in developing countries can strongly influence wives’ choices in health-seeking behavior. Husbands may limit the mothers from getting treatment. Women described a financial barrier as a factor for not getting treatment. This is also related to the lack of support from her husband.

*One of the mothers stated that fear of keeping children alone at home, lack of husband support and low-income limits women from getting treatments*

*“If they have children, they believe that if they come to health facility their children at home will not be safe. Husbands might limit the woman from going to health facilities by telling her she is normal. There is a husband that limits his wife from using family planning. They didn’t come even for ANC check-ups unless they get permission from their husband. Their income will also limit them. She might not have money even for transport and other expenses. I think these are the reasons” (Participant 13, 29-year-old woman).*

### Suggested advice for mothers, families, and health facilities

Study participants forwarded plenty of ideas on the way that pregnant mothers suffering from raised blood pressure and convulsion can access and receive sufficient treatment at the earliest point. These include ANC check-ups regularly, counseling & encouraging mothers, coming to a hospital at the earliest point, husband support, receiving advice only from health professionals, support from families and health professionals, and arranging referrals for the complicated cases.

One of the study participants said that mothers should go regularly for ANC check-ups, and the need to counsel, inform, and encourage mothers.

*“The mother should go regularly for ANC check-up. We [other community members] need to inform, counsel, and encourage mothers to come to a health facility. The government and families should encourage the mothers to visit health facilities when they develop symptoms related to hypertension or other health problems” (Participant One, 27-year-old woman).*

The other study participants also said mothers should come to a hospital at the earliest point and families and health professionals should help them.

*“If she didn’t lose her consciousness, she needs to say bring me to hospital. She needs to come to the hospital earlier. It’s really difficult if her family didn’t encourage and support her [women]. That will be good if she lacks interest than if they lack interest in going to a health facility. They need to treat mothers. They need to give drugs on time” (Participant 10, 20-year-old woman).*

Another mother also shared a similar idea with the above study participants.

*“Firstly, mothers need to get ANC check-ups. They need to have regular ANC check-ups. They will know their status when they have ANC check-ups. Besides, their families need to support them. Families need to counsel them to avoid worry. Families need to encourage her and fulfill all required things. Health facilities need to provide all necessary treatments. They need to provide required treatment” (Participant 13, 29-year-old woman).*

Similarly, the other mother said that health facilities should refer cases beyond their capacity.

*“Health facilities need to treat the mothers. However, if it is beyond their capacity, they need to transfer the mother to the best set-up” (Participant 15, 26-year-old woman).*

One of the study participants also raised husbands should support their wives and mothers should receive information from health care providers.

*“Every husband, whether educated or uneducated, needs to support his wife. Mothers should receive information from health professionals. The doctors need to respect and give due attention for the baby for we don’t know the destiny of that baby” (Participant 16, 20-year-old woman).*

## Discussion

The current study explored the experiences of women who suffered and survived pre-eclampsia and eclampsia in the public hospitals of Shashemene Town, within the Oromia Region, of Ethiopia. The emerged themes in the current study include antenatal care services received and their quality, awareness on raised blood pressure and or convulsion during pregnancy, symptoms experienced, and quality of care received, women’s reaction towards the diagnosis, barriers, and suggested advice to improve health care services received by women who developed pre-eclampsia and eclampsia.

Counseling received during antenatal care is one of the most significant markers of quality services for antenatal care. Women’s narratives in this regard showed that during their antenatal check-ups for the current pregnancy, they did not get counseling. According to Hutchinson, women in their study received inadequate preeclampsia education throughout the antenatal period, and they believed that this was a significant factor in the delays that women with preeclmapsia had when trying to get treatment for their disease. ^11^

The present study showed that women’s level of awareness on raised blood pressure and or convulsion occurring during pregnancy was modest and poor. Most of the women were not aware that pregnant women may suffer from raised blood pressure and convulsion. The current finding is in line with the study conducted in Brazil by Lima de Souza N *et al*. which revealed unawareness of women about eclampsia during prenatal care. They only became aware after hospitalization or by the imminent premature delivery. ^9^

This finding is in line with a study carried out in Nigeria where only a few study participants associate eclampsia with raised blood pressure. ^12^^,^^13^ Similarly, the finding is consistent with the study conducted in Ghana which reported a high prevalence of inadequate knowledge of pre-eclampsia among the pregnant study population. ^14^

Women’s naratives in the present study revealed that raised blood pressure can cause maternal and fetal death. This finding is consistent with the study done in Southwestern Uganda which showed that participants were aware of the potential consequences of the symptoms of eclampsia and mentioned that the condition could lead to the death of the baby in utero or even the mother. ^15^ Similarly, the present study is in line with the study done in India in which study participants mentioned the most serious and most commonly cited consequences of hypertension or seizures in pregnancy to be the death of the mother and/or infant. ^16^

Participating women’s narratives in the present study revealed that mothers might not have money even for transport and other expenses. This was one of the factors that limit pregnant women who developed pre-eclampsia and eclampsia from accessing and getting treatment from health facilities. This finding is supported by the recent study carried out among survivors of pre-eclampsia and eclampsia in which financial barriers affected access to transportation and affordability of health services or medicines. ^16^

Women also mentioned misconception as a barrier for not getting treatment at the earliest point. They stated that swelling of the face & feet during pregnancy is thought to be normal within socio-cultural context. They believe that blood is accumulated in the face & feet till childbirth, and hence they are swollen. Similarly, in a study conducted in Nigeria, it was found that spirituality and tradition affected women’s lives profoundly, which has significantly affected care-seeking decisions. ^10^ Such misconception in society may delay mothers or make them stay at home which may result in life-threatening complications.

Women’s discourses in our study also revealed that unsupportive husbands are barriers to not getting treatment. Husbands are mostly the only financial contributor to a family, and thus the single decision-maker in a family. Therefore, if they are uncooperative, they can significantly delay healthcare seeking or forbid the mothers from going to health facilities. This finding is supported by a study carried out in Nigeria ^10^ & Bangladesh. ^17^

In the present study, women were often not counseled on danger signs, and, as a result, they didn’t know these signs when they experienced them. Few women mentioned that women who have raised blood pressure can’t stand, have headaches on one side, and blurring of vision. But, in the study carried out in India women reported signs or symptoms of pre-eclampsia were sweating, fatigue, dizziness-unsteadiness, swelling, anger, talking too much, frothing, shaking, eyes rolling upwards, clenching teeth, and tongue biting. ^16^

Women’s narratives showed that they were very concerned when they were told that they had hypertension, which they described to be so dangerous. Study subjects also expressed that it is difficult to have raised blood pressure and it will also result in depression.

The present finding is consistent with the study done in Australia where understandably, women were very concerned about the life-threatening nature of pre-eclampsia and focused their attention on the consequences of pre-eclampsia across different end-organs, including brain, liver, and kidneys. ^18^

The present study is also in line with the study in which women described that they were afraid of this disease because they thought that not only themselves but also their babies were at risk and even death and also, they had negative emotions and thoughts when suffering pre-eclampsia. Sadness, frustration, or low-spiritedness were their usual emotions. ^19^

Participating women’s narratives suggested that every husband, whether educated or uneducated, needs to support her to enhance access to health care services. Besides, they described the importance of support from families & society as a key tactic for mitigating complications from pre-eclampsia & eclampsia. This finding is in line with the study done in Nigeria which showed that engaging in discussions with family and communities emerged as a critical cue to action as these groups often provide instrumental support in women’s ANC and delivery care-seeking. ^10^

Women’s naratives suggested the importance of regular ANC check-ups to avert complications of pre-eclampsia and eclampsia. ANC allows early detection & management of complications. They also mentioned the importance of counseling on danger signs of pregnancy during ANC check-ups. This finding is supported by a study conducted in Indonesia which revealed that antenatal care visits affect early detection of pre-eclampsia, and pregnant women who regularly make antenatal visits can prevent possible dangers of pregnancy as early as possible. ^20^

## Conclusion

The current study showed that women lacked adequate knowledge about preeclampsia and eclampsia. Besides, misconceptions about the disease, failure to get counselling on danger signs of pregnancy, not getting standard ANC and being scared and frustrated about the disease are some of issues experienced by the women. It is crucial to raise community leaders’ and health extension workers’ awareness of hypertensive disorders in pregnancy, improve the standard of ANC checkups, and increase the counseling services offered to women attending antenatal care regarding pregnancy danger signs. Encouraging husbands to involve in the care of their wives is also invaluable. The community, families & health care providers are expected to provide all the required support & care for women with pre-eclampsia & eclampsia.

## Ethical considerations

Ethical approval was taken from Madda Walabu University, Shashemene campus, research, and community service coordinating office (reference number: R/C/S/T/T/014/13). Formal letters were obtained from officials of respective hospitals. In addition, informed verbal consent was obtained from study participants because the study participants were uneducated, and this was approved by Institutional Review Board (IRB) of the University. The consent form were translated in to local language and data collector read out for the respondents and took consent before commencing data collection. The consent was narrated to the participants and their responses were tape-recorded. Respondents were notified about their right to refuse or terminate at any point of the interview. The information that was provided by each respondent was kept confidential. All personal identifiers were removed from transcripts and quoted texts. In addition, the Institutional Review Board (IRB) of the University approved the pre-testing of the instruments at Negelle Arsi Primary Hospital and protected the rights of the study participants.

## Data Availability

Zenodo: Lived experiences of women who survived from pre-eclampsia and eclampsia in Public Hospitals of Shashemene Town, Oromia Region, Ethiopia: a qualitative study, https://doi.org/10.5281/zenodo.7576222. ^21^ This project contains the following underlying data:
- Negeso project.atlcb (which contains primary translated document, quotes and codes) This project contains the following extended data:
- S1 Interview Guide Question.docx. Data are available under license Creative Commons Attribution 4.0 International (CC BY 4.0).
