# Supplementary material for: Lived experiences of women who survived from pre-eclampsia and eclampsia in public hospitals of Shashemene Town, Oromia Region, Ethiopia: a qualitative study
Source: F1000Res. 2025 Apr 7;12:1464. Originally published 2023 Nov 13. [Version 3] doi: 10.12688/f1000research.129648.3 (PMC12134722; doi:10.12688/f1000research.129648.3)
Supplement: Peer review history

# Reviewer report: Reviewer response for version 3

**DOI:** 10.5256/f1000research.180022.r382244

**Published:** 2025-06-03

**Authors:** Ira Bernstein

**Affiliations:** 1 University of Vermont, Burlington, Vermont, USA

 This study by Gebeyehu et al is a descriptive study performed via purposive sampling to preeclampsia/eclampsia patients in Ethiopia to explore their experience, understanding, and barriers to care for their pregnancy complication. This is an important study that examines factors that greatly influence patient outcomes, both short- and long-term after complex pregnancies.  The study design is appropriate, including 17 women, 12 with preeclampsia and 5 with eclampsia, cared for in two hospitals who underwent individual interviews, as adapted from a prior study also conducted in Africa. Responses were coded by 3 reviewers using both verbal data via audio recording and non-verbal responses as noted during the interview, with additional review for conformability.

 The data from patient narratives highlighted a need for improvement in education on and routine assessment for signs/symptoms of HTN in pregnancy, education at time or and after patients' diagnoses, a lack of trust in the provider-patient relationship, and a need for self-advocacy to receive appropriate care. Barriers to treatment were appropriately highlighted, emphasizing areas for improvement which would likely influence rate/timeliness of seeking and accessing care among the patient population examined. 

 I am impressed by the authors' pursuit of understanding such a fraught and important topic from the patient perspective. I do believe their findings are of interest for dissemination. I would recommend additional revision, perhaps by a native English speaker, both to improve grammar and ensure appropriate utilization of medical phrases in the article prose and interview translations to English. For example, a "termination" was referred to under "Women's reaction after informed of the diagnosis" though it seems this was referring to a delivery of a preterm infant rather than ending a pregnancy with the intention not to deliver a viable infant.

 An expansion in the number of subjects could highlight if dominant and recurrent specific issues in clinical care could be addressed.

  am not clear how a sample size determination in made for qualitative data? Perhaps this section could be expanded

 It would be of value to have a more detailed outline of the prenatal care environment (ANC) for these subjects i.e. frequency of visits, training of the prenatal care providers, location of visits?

Is the work clearly and accurately presented and does it cite the current literature?

Yes

If applicable, is the statistical analysis and its interpretation appropriate?

Not applicable

Are all the source data underlying the results available to ensure full reproducibility?

Partly

Is the study design appropriate and is the work technically sound?

Partly

Are the conclusions drawn adequately supported by the results?

Partly

Are sufficient details of methods and analysis provided to allow replication by others?

Yes

Reviewer Expertise:

Preeclampsia physiology and prepregnancy predisposition, Preeclampsia follow-up and cardiometabolic risk

I confirm that I have read this submission and believe that I have an appropriate level of expertise to confirm that it is of an acceptable scientific standard, however I have significant reservations, as outlined above.

# Reviewer report: Reviewer response for version 2

**DOI:** 10.5256/f1000research.170549.r319023

**Published:** 2024-09-19

**Authors:** Margaret P. Kasaro

**Affiliations:** 1 Division of Global Women's Health, Department of Obstetrics and Gynecology, The University of North Carolina at Chapel Hill, Chapel Hill, North Carolina, USA

I have reviewed the revised version of the paper and happy with the progress so far. There however may still be need to for further revision eg: 

 Conclusion in abstract could be edited to not imply that quality of care is poor but that it was reported so

 The data collection section of methodology still has details that may not need to be in the final write up:

 Pretesting details

 Line 2 in paragraph 3 of data collection could be deleted

 Data collection details

 Transcription details

 Consider if the last paragraph in data collection is needed 

 Conclusion is still too long and not summarised to deliver message effectively

 In general review for English usage may be beneficial

Is the work clearly and accurately presented and does it cite the current literature?

Yes

If applicable, is the statistical analysis and its interpretation appropriate?

Yes

Are all the source data underlying the results available to ensure full reproducibility?

Yes

Is the study design appropriate and is the work technically sound?

Yes

Are the conclusions drawn adequately supported by the results?

Partly

Are sufficient details of methods and analysis provided to allow replication by others?

Yes

Reviewer Expertise:

Women's healthHmaternal and child health

I confirm that I have read this submission and believe that I have an appropriate level of expertise to confirm that it is of an acceptable scientific standard, however I have significant reservations, as outlined above.

# Response

**Published:** 2025-03-28

**Authors:** Negeso Gejo

**Affiliations:** Madda Walabu University, Ethiopia

**Responses to Reviewer:**

 We would like to appreciate Margaret P. Kasaro, for reviewing the second version of the article. We would like to extend our gratitude her constructive comments and presented our point by point responses as follows:

 Comment 1: Conclusion in abstract could be edited to not imply that quality of care is poor but that it was reported so

 Response: We have refined it as per the comment.

 Comment 2: The data collection section of methodology still has details that may not need to be in the final write up: Pretesting details: Line 2 in paragraph 3 of data collection could be deleted. Data collection details, Transcription details. Consider if the last paragraph in data collection is needed

 Response: During the initial process of the review process, editors form journal gave due attention on the methodology section and recommended the authors to write details of methodology section including the data collection process and analysis. That’ why we write up the aforementioned issues in details. It’s known that every scientific paper has to be done as per the standard. Every steps that was gone to ensure the quality of the research has to be clearly written and communicated to the scientific community. Thus, we kindly request the reviewer to consider the above issue.

 Comment 3: Conclusion is still too long and not summarised to deliver message effectively.

 Response: We have summarized it as per the comment.

# Reviewer report: Reviewer response for version 2

**DOI:** 10.5256/f1000research.170549.r319024

**Published:** 2024-09-11

**Authors:** Marianne Johansen

**Affiliations:** 1 Department of Obstetrics, Rigshospitalet, Copenhagen University Hospital, Copenhagen, Denmark

I still believe that this paper needs a thorough proof reading by a native English speaking person. Also some grammar and spelling mistakes exist in the paper.

 The Methods section is still somewhat unclear and too detailed. The reference to the Nigerian study (reference 10) does cover some of the text that could be shortened. Besides, I believe it would be of interest to the reader to know how many women were invited to participate in order to include 17 interviews.

 In my opinion the manuscript still contains too many repetitions – both regards content and text. Also too many themed quotes are included in the paper not all allowing for new insight.  

 In the “Emerged themes” section the authors write “ One woman”, “Another woman”, “One of the participant”, “the above woman” and many more ways to characterize the participants. I suggest that they use participant numbers instead to simplify.

 Table 1 is fine

 Reviewer 1 comment no 4 has not been addressed sufficiently in this revised version of the paper.

 Good luck with the final revisions of the paper.

Is the work clearly and accurately presented and does it cite the current literature?

Partly

If applicable, is the statistical analysis and its interpretation appropriate?

Not applicable

Are all the source data underlying the results available to ensure full reproducibility?

Partly

Is the study design appropriate and is the work technically sound?

Yes

Are the conclusions drawn adequately supported by the results?

Yes

Are sufficient details of methods and analysis provided to allow replication by others?

Yes

Reviewer Expertise:

Preeclampsia

I confirm that I have read this submission and believe that I have an appropriate level of expertise to confirm that it is of an acceptable scientific standard.

# Reviewer report: Reviewer response for version 1

**DOI:** 10.5256/f1000research.142346.r304794

**Published:** 2024-08-16

**Authors:** Margaret P. Kasaro

**Affiliations:** 1 Division of Global Women's Health, Department of Obstetrics and Gynecology, The University of North Carolina at Chapel Hill, Chapel Hill, North Carolina, USA

Hypertensive disorders in pregnancy are an important contributor to maternal morbidity and mortality. This is a write up on the experiences of the women who survived preeclampsia and eclampsia for knowledge of  symptomatology, antenatal services - information and education on danger signs, referral and management of the disorder. The study has highlighted gaps in the health care delivery as experienced by the women who survived the disorder but in conclusion has not included the implications and recommendations in working towards effective engagement. Here are few comments to consider for the next version of the paper:

 Strengths and limitations

 1.The study design may need to be reconsidered to be mentioned as a strength of there study

 2. If the translation process was rigorous to ensure the meaning is not lost in translation, can this point still be a limitation

 3. Selection bias exists not exits

 Introduction

 1. Does the use of the words commonly and usually in the definition of Preeclampsia affect the meaning here?

 2. Paragraph 2, 3 and 4 are repetitious in the discussion of effects of PE and eclampsia please edit

 3. Paragraph 5 there is repeating of a sentence. In editing consider including how data on experiences may be used to reduce the negative effects of the disease

 Sample size calculation

 1. It may not be necessary to explain when to stop sampling plus there is repeating

 Data collection

 1. Please note the repetition of sampling techniques description

 2. Naming the staff who were actually doing the data collection should be reserved for an appropriate section

 3. Last sentence in paragraph 1 is not clear

 4. In paragraph 2  please consider the following:

- You have described the translation process, is it rigorous not to have information lost in the process?
- The relationship between confidentiality  and discomfort
- Indicate that open ended questions and probing were used  rather that specific  questions
- Is collection to saturation equal to credibility 
- Data collected is collectors 
- In the last sentences - which technique is being referred to for the nonverbal
- Dependability, transferability and conformability  confirm the meaning in this context

Analysis

 Overall consider editing the analysis section to use standard format of reporting qualitative analysis 

 then consider removing the bold in the sentence … translation by **PI and co investigators**

 Results

- Please pay attention to the tenses
- space between 17 and women
- In sentence number 2 edit to add bold word as follows - the number of children **that** the women

Themes

- Quality of care is repeated
- Please clarify on the suggested pieces of advice

ANC

- Edit the sentence to clarify if each participant reported different care or is it their interpretation that care offered to women varies
- Last sentence suggested edit - Services that women reported to have received included...…
- Women requested to give opinions on the quality …. by data collectors may imply the care was provided by the data collectors

Women's reactions

 Consider not explaining what procedure to terminate means

 Barriers

- Repeating meaning of daataa
- Consider rephrasing the unsupportive husbands and low income/advice preamble

Discussion

- Paragraph one summarises the findings well
- Tackle each theme in order of how presented in paragraph 1
- Women's discourse could be edited with synonyms

Conclusions

 Please consider editing the conclusion without repeating results in detail and put in implications and recommendations

Is the work clearly and accurately presented and does it cite the current literature?

Yes

If applicable, is the statistical analysis and its interpretation appropriate?

Yes

Are all the source data underlying the results available to ensure full reproducibility?

Yes

Is the study design appropriate and is the work technically sound?

Yes

Are the conclusions drawn adequately supported by the results?

Partly

Are sufficient details of methods and analysis provided to allow replication by others?

Yes

Reviewer Expertise:

Women's healthHmaternal and child health

I confirm that I have read this submission and believe that I have an appropriate level of expertise to confirm that it is of an acceptable scientific standard, however I have significant reservations, as outlined above.

# Reviewer report: Reviewer response for version 1

**DOI:** 10.5256/f1000research.142346.r250739

**Published:** 2024-03-21

**Authors:** Marianne Johansen

**Affiliations:** 1 Department of Obstetrics, Rigshospitalet, Copenhagen University Hospital, Copenhagen, Denmark

This exploratory qualitative study by N Gebeyehu et al on the lived experiences of 17 women who have all experienced preeclampsia or eclampsia in two public Ethiopian hospitals - one general and one referral hospital - is generally an important and well conducted study.

 The authors and investigators address and explore some very important issues on different aspects like levels of awareness of the preeclampsia syndrome in the general population and at a healthcare level in the ANC, misconceptions in the society, barriers to access to healthcare and the the level of quality of counselling and healthcare provided.

 Importantly, the interviewed women bring suggestions to the table on how healthcare counselling and better access to qualified healthcare may improve the clinical outcome in the region for women suffering from preeclampsia.

 These are all very essential issues to address in order to better inform healthcare workers on how to improve counselling of women in Ethiopia on preeclampsia and eclampsia; both still potentially severe and life threatening conditions for both mother and child.

 I would like to commend the authors for addressing this very important topic as preeclampsia and eclampsia is still one of the leading causes of maternal mortality in the Sub-Saharan African countries.

 However, I have some general and some more specific comments in relation to the submitted paper.

 General comments: 

 Generally a well written manuscript. However, I find that the manuscript is too long and with many repetitive quotes in the result section. One suggestion would be to put the quotes in themed boxes for each of the respective emerged themes; Antenatal care service offered and its quality, Symptoms experienced and quality of care received, Barriers to receiving treatments from health facilities, Misconceptions, lack of knowledge, Unsupportive husband and low income, and Suggested advice for mothers, families and health facilities. 

 I also suggest that the overall number of quotes appearing in the manuscript is reduced, as several of the quotes are very alike from a thematic perspective. 

 Besides, the manuscript would benefit from a thorough proof reading by a native English speaking person. 

 Specific comments: 

 Women are quoted for saying that access to health care services is lacking. In this context it would be relevant to mention whether prescribed medicine is free of charge?

 I am curious to know whether women who participated did in any way differ from women who rejected to participate. Also, I would like to know how many women were invited to participate to allow for 17 participants? 

 5/17 of the participants suffered eclampsia. Is this a normal proportion amongst women with preeclampsia in Ethiopia? Please specify.

Is the work clearly and accurately presented and does it cite the current literature?

Partly

If applicable, is the statistical analysis and its interpretation appropriate?

Not applicable

Are all the source data underlying the results available to ensure full reproducibility?

Partly

Is the study design appropriate and is the work technically sound?

Yes

Are the conclusions drawn adequately supported by the results?

Yes

Are sufficient details of methods and analysis provided to allow replication by others?

Yes

Reviewer Expertise:

Preeclampsia

I confirm that I have read this submission and believe that I have an appropriate level of expertise to confirm that it is of an acceptable scientific standard, however I have significant reservations, as outlined above.
